# Accuracy of prediction models for long-term type 2 diabetes remission after gastric bypass

**DOI:** 10.1007/s00592-023-02092-1

**Published:** 2023-04-21

**Authors:** Samuel Cardoso, Sofia S. Pereira, Rui F. Almeida, Catarina Osório, Diogo Silva, Mário Nora, Mariana P. Monteiro, Marta Guimarães

**Affiliations:** 1 ICBAS - Instituto de Ciências Biomédicas Abel Salazar, UMIB-Unidade Multidisciplinar de Investigação Biomédica, Universidade Do Porto, Porto, Portugal; 2 ITR-Laboratory for Integrative and Translational Research in Population Health, Porto, Portugal; 3 Department of General Surgery, Centro Hospitalar de Entre O Douro E Vouga, Cândido Pinho, 4520-211 Santa Maria da Feira, Portugal; 4 Department of Anatomy of Institute of Biomedical Sciences Abel, Salazar - University of Porto, Jorge Viterbo Ferreira 228, Building 1.3, 4050-313 Porto, Portugal

**Keywords:** Type 2 diabetes remission, Roux-en-Y gastric bypass, Prediction models, Type 2 diabetes

## Abstract

**Aim:**

To evaluate the accuracy of DiaBetter, DiaRem, Ad-DiaRem and 5y-Ad-DiaRem scores’ at predicting T2D remission 10 or more years after surgery.

**Methods:**

Patients with obesity and T2D (*n* = 126) submitted to RYGB with 10 or more years of follow-up. It was a unicentric trial. Pre-operative anthropometric and clinical data was retrieved to calculate DiaRem, DiaBetter, Ad-DiaRem and 5y-Ad-DiaRem scores, while a hospital visit was conducted to assess current diabetes status. The area under the receiver operating characteristic (AUROC) curve was calculated as estimate of the scores’ accuracy to predict long-term T2D remission.

**Results:**

Among the entire cohort (*n* = 126), 70 subjects (55.6%) achieved and maintained T2D remission 10 or more years after RYGB. The 5y-Ad-DiaRem score was the one that depicted the highest discriminative power (AUROC = 0.838) to predict long-term T2D remission when compared to DiaBetter (AUROC = 0.735), DiaRem (AUROC = 0.721) and Ad-DiaRem (AUROC = 0.720).

**Conclusion:**

The score with highest accuracy to predict long-term T2D remission after RYGB surgery was the 5y-Ad-DiaRem. Yet, the available scores accuracy to predict T2D remission in the long term is still suboptimal, highlighting the unmet need for a better scoring system.

## Introduction

Bariatric surgery is the most effective treatment for severe obesity [1]. Roux-en-Y Gastric Bypass (RYGB) is a widely performed bariatric surgery procedure with a very favourable benefit-to-risk ratio. The vast majority of patients submitted to RYGB experience substantial and sustained weight loss [2], as well as, significant improvement or resolution of obesity-associated comorbidities, such as Type 2 Diabetes (T2D) and metabolic syndrome [3–6]. Nevertheless, within the first two years after surgery 5–15% of the patients fail to achieve clinically relevant weight loss or diabetes remission [7]. A percentage that tends to increase along the timespan after surgery with an estimated long-term failure rate of 20–35% [8, 9]. This figure was corroborated by our previous study that evaluated the weight loss and comorbidities remission rate 10 or more years after RYGB, which allowed to demonstrate that less than one-third of the patients did not achieve the weight loss goal and only 54.2% of patients achieved T2D remission [10].

Several clinical and biochemical patient features have been so far identified as predictors of T2D remission. Major determinants include the magnitude of preserved pancreatic function and peripheral insulin resistance reduction [11]. Thereby, preoperative features such as advanced age, higher body mass index (BMI), longer T2D duration, greater glycated haemoglobin (HbA1c), higher fasting glucose levels, lower C-peptide levels and use of insulin therapy tend to be associated with lower probability of T2D remission after bariatric surgery [12, 13]. In particular, a previous study found that a preoperative HbA1c < 7.1% have a sensitivity of 85% to predict T2D remission at 1 year after surgery [14]. Additionally, a greater total weight loss (%TWL) after surgery tends to be a positive predictor of T2D remission [15].

Based on the above mentioned determinants of T2D remission, several predictive models have been devised, such as DiaBetter [16], DiaRem [17], Ad-DiaRem [18] and 5y-Ad-DiaRem [19]. These mathematical models were demonstrated to predict T2D remission in the short-term, with an accuracy that ranges between 75 and 90% for RYBG [13, 16, 18, 20, 21]. Additionally, the 5y-Ad-DiaRem score used to predict medium-term T2D remission, includes not only preoperative determinants but also weight loss 1-year after surgery [19]. Anyhow, the accuracy of the above mentioned scores to predict T2D remission tends to decrease along follow-up time, to under 75% at medium term, i.e. 5 or more years after surgery [22, 23], while the accuracy of these models at predicting long-term T2D remission has not been reported.

Thus, the aim of the present study was to evaluate the accuracy of DiaBetter, DiaRem, Ad-DiaRem and 5y-Ad-DiaRem scores at predicting long-term T2D remission, i.e., 10 or more years after RYGB surgery.

## Methods

### Patient’s and methods

**Fig. 1:**
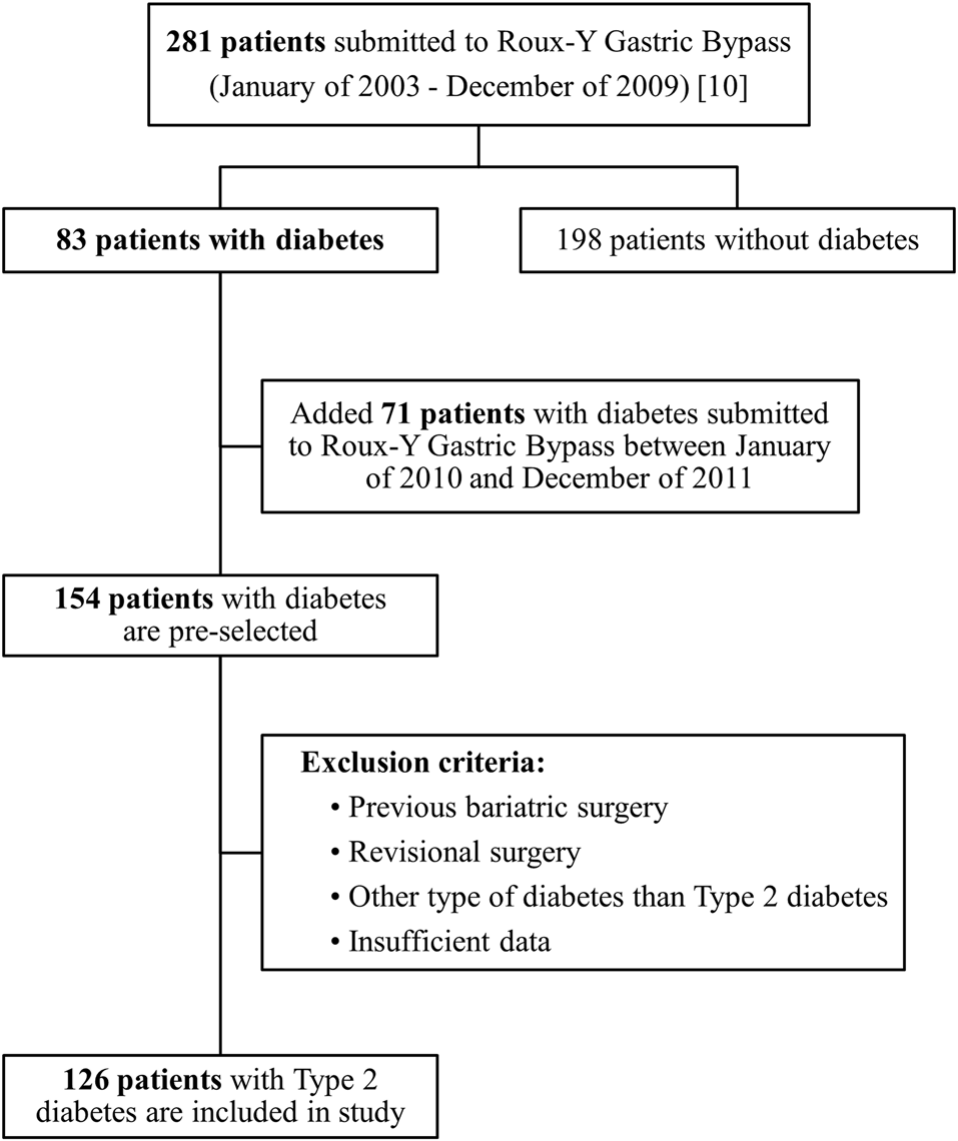
Flowchart of the study

This was a single centre retrospective observational cohort study. Data from patients with obesity (BMI ≥ 35 kg/m^2^) submitted to RYGB, between January of 2003 and December of 2009 for obesity treatment, who completed 10 or more years of follow-up after surgery, were retrieved for analysis. From the initial cohort of 281 patients, 83 had a pre-operative diagnosis of diabetes [10]. This initial cohort was later expanded by 71 additional patients with type 2 diabetes submitted to RYGB between January of 2010 and December of 2011, who at the time of the initial cohort recruitment, had not yet completed 10 years of follow-up after RYGB. Thereby, 154 patients were pre-selected to be enrolled in this study. Patients with a previous bariatric intervention who underwent revisional surgery, who had a diagnosis of diabetes other than type 2, such as type 1 diabetes or Latent Autoimmune Diabetes of the Adult (LADA) or did not have enough data available to calculate at least one of the four prediction scores, were excluded from the study. Consequently, 126 patients were included in this study for statistical analysis (Fig. 1).

Preoperative clinical evaluation consisted of a full medical history, physical examination and fasting blood analysis. The parameters evaluated were age, body weight, BMI, comorbidities, ongoing drugs, fasting blood glucose and HbA1c.

All patients underwent bariatric surgery at a single centre by the same team of bariatric surgeons. The surgical procedure consisted in a standard RYGB procedure with a constant alimentary limb length (120 cm) and a variable biliopancreatic limb length (50–200 cm) [24].

After surgery, patients were followed up by a multidisciplinary team (bariatric surgeon, endocrinologist, psychologist and registered dietitian) attending regular outpatient hospital visits for the first three years after surgery, and thereafter by the general practitioner. Routine hospital visits included sequential revaluation of body weight and fasting blood analysis that included the measurements of glucose and HbA1c.

Patients were considered as being in T2D remission, at 1-year and 10 or more years after surgery, if HbA1c was under 6.5% without use of any antidiabetic drug and as having diabetes relapse whenever HbA1c was above 6.5% with use of any antidiabetic drug after experiencing a period of remission [25].

Preoperative anthropometric and clinical data, including age, HbA1c, T2D duration and the number and type of antidiabetic drugs, were used to calculate DiaBetter [16], DiaRem [17], Ad-DiaRem [18] scores. Among the 3 diabetes remission prediction scores, DiaRem is the only one that does not takes into consideration T2D duration, while Ad-DiaRem considers not only T2D duration but also the number of antidiabetic drugs used, and DiaBetter score is the most simple score to calculate, as it only considers 3 parameters (HbA1c, T2D duration and the type of antidiabetic medication) weighted on a scale between 0 and 3. For the 5y-Ad-DiaRem [19] score, calculations required a combination of preoperative and postoperative 1-year parameters, namely number of antidiabetic drugs, fasting blood glucose, body weight lost and 1-year T2D remission status.

### Calculations and statistical analysis

Anthropometric data were used to calculate Body Mass Index (BMI, calculated as, [weight (Kg) ÷ height^2^ (m^2^)]), percentage of total weight loss (% TWL, calculated as [[(preoperative weight–postoperative weight) ÷ (preoperative weight)] × 100]) and percentage of excess weight loss (% EWL, calculated as [[(preoperative weight–postoperative weight) ÷ (preoperative weight–(25 × height^2^ (m^2^)))] × 100]).

Categorical variables are expressed as number of cases and percentage (%), and the quantitative variables are expressed as mean and standard deviation. Categorical variables were analyzed by the *χ*^2^ test. To compare three or more independent experimental groups, we used the one-way ANOVA for normally distributed variables and the Kruskal–Wallis for variables that did not present a normal distribution.

The diagnostic accuracy of these scores for predicting long-term T2D remission was evaluated using the receiver operating characteristic (ROC) curve. ROC curves were used to compare the accuracy of prediction models (DiaRem, DiaBetter, Ad-DiaRem and 5y-Ad-DiaRem) between the different T2D remission groups: patients with T2D remission (10y-DR), patients that never experienced T2D remission during the 10 years (10y-NDR), patients that experienced T2D remission but relapsed (10y-Relapse) and patients with T2D at 10 years (non-remission group) that merges the 2 previous groups. In a ROC curve, the true positive rate (sensitivity) is represented as a function of the false positive rate (1-specificity) for different cut-off points of a parameter. The area under the ROC curve (AUROC) was used to measure how accurate is a score at predicting T2D remission. Based on the AUROC, the test can be considered excellent between 0.90 and 1.00; good between 0.80 and 0.90; fair between 0.70 and 0.80; and poor between 0.60 and 0.70; and failed for values below 0.60.

A *p-value* < 0.05 was considered statistically significant. All statistical analyses were performed with the aid of the GraphPad Prism software version 8.0 and IBM SPSS Statistics version 27, both for Windows.

## Results

**Fig. 2:**
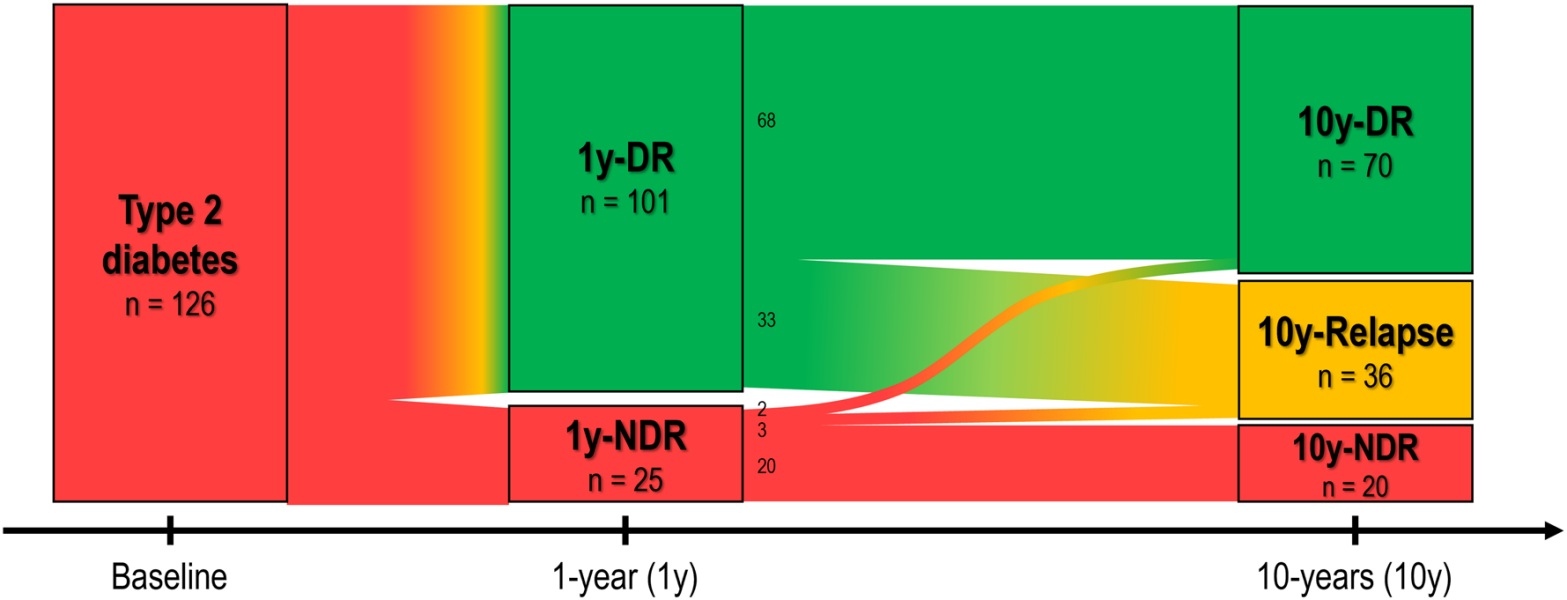
Evolution of T2D status in 126 patients of the cohort study, throughout the follow-up, 1 and 10 years after RYGB. T2D remission was considered if HbA1c was below 6.5%, in the absence of any antidiabetic drug. Abbreviations: 1y-DR – Diabetes remission in the first year after RYGB; 1y-NDR – No diabetes remission in the first year after RYGB; 10y-DR – Diabetes remission group at 10 or more years after RYGB; 10y-Relapse – Relapse group at 10 or more years after RYGB; 10-NDR – Never diabetes remission group at 10 or more years after RYGB

The long-term outcomes of a cohort of 126 patients with obesity and T2D submitted to laparoscopic RYBG at a single centre were assessed. Of these patients, 80.2% experienced T2D remission 1 year after surgery, whereas the rate of T2D remission decreased to 55.6% (*n* = 70) at 10 years after RYGB. Therefore, 33 patients (32.7%) with T2D remission in the first year after surgery (1y-DR), were found to have relapsed 10 years after RYGB (Fig. 2).

Afterwards, clinical and biochemical parameters of patients with different trajectories: 10y-DR, 10y-Relapse and 10y-NDR were compared (Table 1).

**Table 1:** Baseline and post-operative characteristics of the study patient cohort

Variable	10y-DR (*n* = 70)	10y-Relapse (*n* = 36)	10y-NDR (*n* = 20)	*p-value*
*Before surgery*				
Female, *n* (%)	60 (85.7%)	31 (86.1%)	18 (90.0%)	0.882
Age (years)	48.9 ± 8.8	47.5 ± 6.5	51.6 ± 6.7	0.180
Weight (kg)	112.9 ± 16.9	109.8 ± 16.4	108.7 ± 13.7	0.484
BMI (kg/m^2^)	44.0 ± 6.0	42.6 ± 5.6	43.2 ± 5.1	0.483
35 ≤ BMI ≤ 40	19 (27.1%)	14 (38.9%)	7 (35.0%)	0.768
40 < BMI ≤ 45	27 (38.7%)	11 (30.6%)	7 (35.0%)	
45 < BMI ≤ 50	12 (17.1%)	8 (22.2%)	3 (15.0%)	
BMI > 50	12 (17.1%)	3 (8.3%)	3 (15.0%)	
Follow-up time (years)	11.3 ± 1.1	11.6 ± 1.1	11.0 ± 1.2	0.380
Hypertension, *n* (%)	52 (74.3%)	26 (72.2%)	19 (95.0%)	0.110
Dyslipidaemia, *n* (%)	38 (54.3%)	25 (69.4%)	15 (75.0%)	0.132
Obstructive sleep apnoea, *n* (%)	7 (10.0%)	7 (19.4%)	3 (15.0%)	0.394
Diabetes characteristics				
Diabetes duration (years)	3.6 ± 3.7 [59]	6.0 ± 5.4 [32]	12.0 ± 9.5 [19]	< 0.001
Insulin therapy, *n* (%)	0 (0%)	5 (13.9%)	8 (40.0%)	< 0.001
Fasting glucose (mg/dL)	129.8 ± 39.2	174.7 ± 69.6	164.7 ± 66.2	< 0.001
HbA1c (%)	6.1 ± 1.1	7.2 ± 1.8	7.8 ± 2.4	< 0.001
Diabetes remission scores				
DiaRem	3.4 ± 2.9	6.7 ± 5.4	11.0 ± 7.5	< 0.001
DiaBetter	2.6 ± 1.8 [59]	4.2 ± 2.2 [32]	5.4 ± 3.0 [19]	< 0.001
Ad-DiaRem	6.6 ± 3.5 [59]	9.1 ± 4.3 [32]	12.8 ± 6.0 [19]	< 0.001
5y-Ad-DiaRem	5.9 ± 4.0 [56]	9.5 ± 3.5 [32]	17.0 ± 3.8 [19]	< 0.001
*After surgery*				
Weight-loss outcomes				
1-month post-RYGB (%TWL)	12.6 ± 4.8 [68]	12.4 ± 3.9	11.0 ± 5.3 [17]	0.425
2-month post-RYGB (%TWL)	17.1 ± 4.8 [67]	16.1 ± 4.6 [35]	15.4 ± 5.5 [16]	0.350
6-month post-RYGB (%TWL)	28.2 ± 5.6 [66]	25.9 ± 5.0 [35]	25.3 ± 7.4	0.055
1-year post-RYGB (%TWL)	33.8 ± 6.8 [69]	30.0 ± 5.3	29.7 ± 8.8	0.007
2-year post-RYGB (%TWL)	34.1 ± 8.2 [65]	28.8 ± 5.6 [33]	28.6 ± 8.1 [15]	0.002
10-year post-RYGB (%TWL)	28.2 ± 10.4	24.0 ± 9.1	27.0 ± 7.5	0.179
1-month post-RYGB (%EWL)	31.1 ± 12.3 [68]	31.9 ± 12.4	28.3 ± 16.4 [17]	0.624
2-month post-RYGB (%EWL)	41.9 ± 13.2 [67]	41.4 ± 14.9 [35]	38.5 ± 17.7 [16]	0.696
6-month post-RYGB (%EWL)	68.3 ± 16.2 [66]	65.7 ± 16.7 [35]	62.7 ± 21.2	0.414
1-year post-RYGB (%EWL)	82.3 ± 18.9 [69]	76.4 ± 17.9	73.8 ± 24.8	0.141
2-year post-RYGB (%EWL)	82.3 ± 20.1 [65]	71.5 ± 15.8 [33]	71.0 ± 22.4 [15]	0.013
10-year post-RYGB (%EWL)	65.3 ± 23.0	59.6 ± 25.0	63.8 ± 21.0	0.329
Diabetes status 1 year after RYGB				
Remission, *n* (%)	68 (97.1%)	33 (91.7%)	0 (0%)	< 0.001

Square parentheses were used if values were not calculated using the total number of patients of subgroup. Abbreviations: 10y−DR – Diabetes remission group at 10 or more years after RYGB; 10y−Relapse – Relapse group at 10 or more years after RYGB; 10y−NDR – Never diabetes remission group at 10 or more years after RYGB; BMI – Body Mass Index; EWL – Excess of weight loss; HbA1c − Glycated haemoglobin; TWL – Total Weight Loss; RYGB – Roux−en−Y Gastric Bypass

These three groups are statistically different in baseline pre-operative T2D related characteristics and in the percentage of body weight loss two years after RYGB. Patients that experienced long-term T2D remission presented a statistically significant shorter duration of diabetes, lower preoperative fasting glucose and HbA1c, and less use of insulin therapy, when compared with other two groups. Although the 10y-DR subgroup had a greater BMI at baseline, patients experienced greater weight loss after bariatric surgery, particularly within the first year after RYGB (%TWL: 33.8 ± 6.8, 30.0 ± 5.3 and 29.7 ± 8.8 *(p* = *0.007)*, for 10y-DR, 10y-Relapse and 10-NDR groups, respectively). Differences in EWL were statistically significant between the three groups at the second year after surgery (%EWL: 82.3 ± 20.1, 71.5 ± 15.8 and 71.0 ± 22.4 *(p* = *0.013)*, for 10y-DR, 10y-Relapse and 10-NDR groups, respectively) (Table 1).

**Fig. 3:**
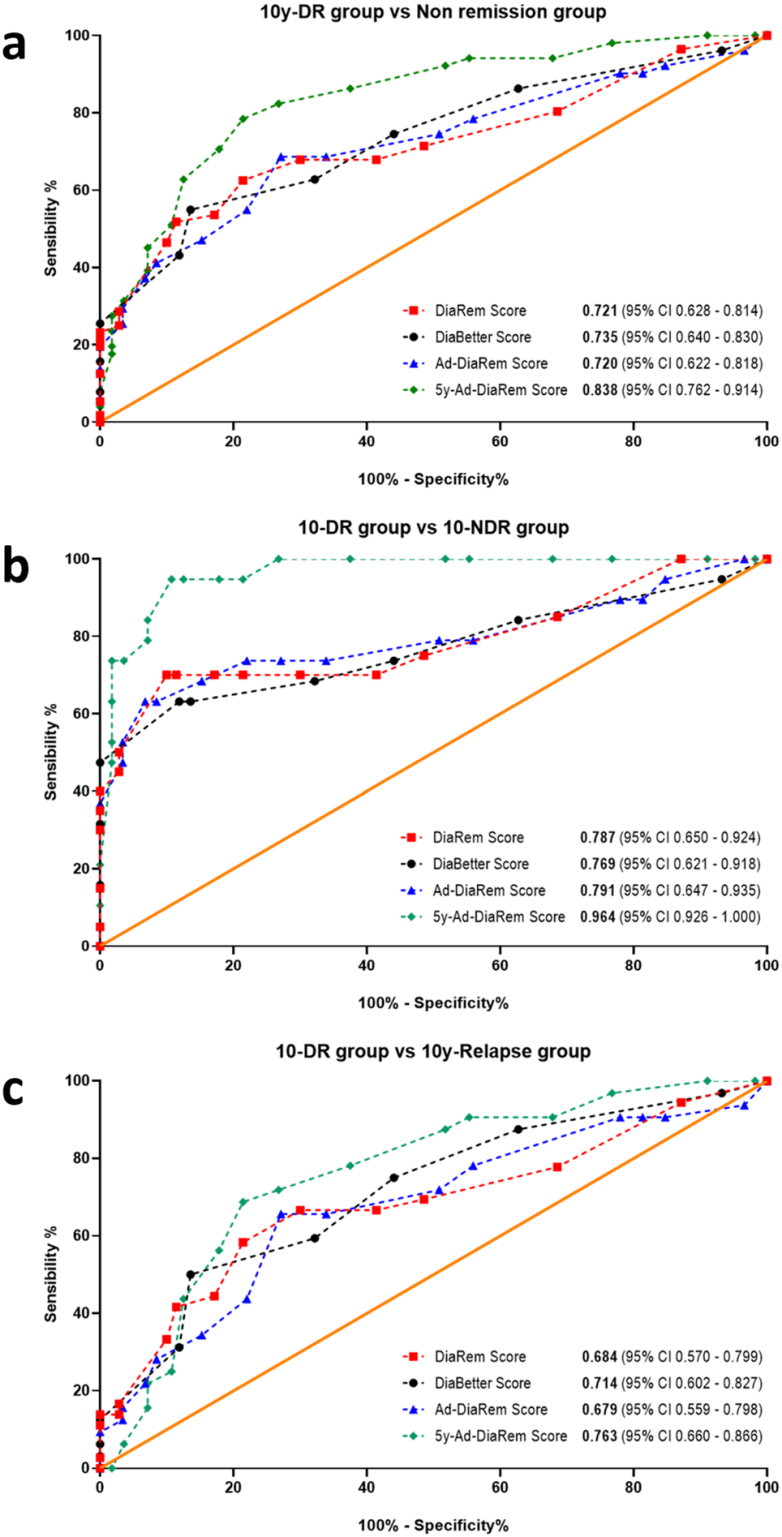
Comparison of the accuracy of prediction models (DiaRem, DiaBetter, Ad-DiaRem and 5y-Ad-DiaRem) in the study population using receiver operating characteristic. (**A**: Comparison between remission and non-remission group (10y-Relapse + 10y-NDR) of T2D at 10 or more years; **B**: Comparison between 10y-DR and 10y-NDR groups; **C**: Comparison between 10y-DR and 10y-Relapse groups). The orange line serves as the reference line

There was a statistically significant difference in the mean AUROC values of T2D remission prediction scores, with an increasing order from 10y-DR to 10y-Relapse and 10y-NDR *(p* < *0.001)*, respectively. The long-term accuracy of T2D remission prediction scores is shown in Fig. 3. When comparing the remission group with the non-remission group (10y-Relapse and 10-NDR), the AUROC was 0.721 for DiaRem, 0.735 for DiaBetter, 0.720 for Ad-DiaRem and 0.838 for 5y-Ad-DiaRem (Fig. 3a). These values increased to 0.787 for DiaRem, 0.769, for DiaBetter, 0.791 for Ad-DiaRem and 0.964 for 5y-Ad-DiaRem, only when 10y-DR and 10y-NDR when compared (Fig. 3b).

## Discussion

Several prediction models can be used to predict the probability of T2D remission in the short term after bariatric surgery. However, the accuracy of these scores at predicting long-term T2D remission was not yet reported. Therefore, the primary aim of the present study was to evaluate the accuracy of pre-stablished prediction scores at predicting long-termT2D remission, i.e. 10 or more years after RYGB.

In the medium and long-term after bariatric surgery, patients tend to experience some degree of weight regain and the percentage of obesity-associated comorbidities resolution decreases, with a reduction in T2D remission rates between the first two years and 10 years after surgery ranging from 28.4 to 32.0% [5, 6]. The present study corroborates these findings, since a T2D remission rate of 80.2% was observed in the first year after RYGB, while the percentage of T2D remission at 10 years after surgery was 55.6%, representing a reduction of 30.7%, of subjects that achieved and maintained T2D remission. A 51.4% reduction in the rates of T2D remission between the second year (72%) and tenth year (35%) was previously reported in the Swedish Obese Subjects (SOS) study [23]. However, this data may not be comparable to the herein study findings, since among the patients included in the SOS study only 17.9% had been submitted to gastric bypass. Furthermore, it is important to highlight the heterogeneity in the diabetes remission criteria adopted by the different studies and across times that hinders comparisons of diabetes remission rates between studies, which should always be carried out with utmost caution [11, 25].

In the herein study, thirty-six patients (28.6%) experienced T2D remission during the follow-up, but had a relapse 10 or more years after RYBG. Similar relapse rates were observed by other authors [26].

The long-term T2D remission in this study was associated with a shorter duration of diabetes, lower preoperative fasting glucose and HbA1c, and less use of insulin therapy before surgery, which are known to be associated with a greater *β*-cell pancreatic function reserve. Therefore, these results support previous findings that T2D duration and C-peptide levels, are among the most powerful predictors of diabetes remission [27–29]. The impact of this biochemical markers is so relevant that C-peptide was included in another diabetes remission prediction score, the ABCD score [30]. However, its widespread use in the clinical setting can be hindered by the fact that C-peptide measurement is not widely available in many routine laboratories, as occurs at our centre. In the absence of C-peptide data, the pancreatic function reserve can still be inferred by the degree of glycaemic control and the need of insulin therapy for glycaemic control prior to bariatric surgery.

T2D outcomes over the long-term after RYBG, beyond pre-operative predictors, is mainly dependent on weight loss within the first year after surgery and weight maintenance over time. Thus, the importance of dietary and lifestyle interventions during the first year after surgery in order to maximize the potential weight loss in order to achieve long-term T2D remission [31], must be stressed and should be a take home message for the clinic. In our study, patients with long-term T2D remission had a TWL in the first year after RYBG 13.0% greater than in patients who presented T2D diagnosis criteria 10 or more years after surgery, and an EWL, at 2 years after surgery, 15.3% greater than long-term T2D no-remission patients.

Comparing the different scores, the 5y-Ad-DiaRem was the one with the highest accuracy at predicting T2D remission at 10 or more years after surgery. Of notice and contrarily to other scores, the 5y-Ad-DiaRem score includes the weight loss and the glycaemic control in the first year after bariatric surgery. Debédat and colleagues when presenting this score defended that pre-operative glycaemic control was more impactful to discriminate patients with T2D remission and without T2D remission 5 years after RYGB. On the other hand, the authors also demonstrated that weight loss in the first year had more influence on the risk of T2D relapse at the end of the same follow-up period [19]. Our current study corroborates the previous findings, given that when comparing patients who did not meet the diagnosis criteria for T2D 10 or more years after surgery with those who did, the 5y-Ad-DiaRem score had a greater accuracy in terms of discriminative power than the other studied scores that only presented a fair accuracy, with values below 0.74.

The accuracy of T2D remission prediction scores increases when the comparison is restricted to 10y-DR and 10y-NDR patients, with the greatest increase for 5y-Ad-DiaRem (AUROC = 0.964). The excellent discrimination power of 5y-Ad-DiaRem, supports the importance of weight loss and the glycaemic control in first year after surgery for the long-term T2D outcomes.

Preoperative prediction of T2D remission can be also useful to address and manage patient expectations on the outcomes of bariatric interventions. Nonetheless, given that a greater weight loss in the first few years after surgery is associated with higher probability of long-term T2D remission, medical and lifestyle interventions in the early post-operative period should be optimized in order to achieve the greatest possible weight loss.

Regardless of the fact that part of the patients never achieve diabetes remission, or experience only a transient remission, there are still significant long-term benefits, which can be translated by a reduction in the needs of antidiabetic drugs, a reduction of Hb1Ac values and a decrease of morbidity and mortality associated with T2D, benefits that the scores do not have the ability to predict [32]. In this study, 41.5% of the patients with BMI > 45 kg/m^2^ did not achieved long-term T2D remission. Therefore, an alternativesurgical procedure, associated with higher rates of diabetes remission and TWL, or an adjuvant medical treatment, after surgery, with antidiabetic drugs, such as metformin, should have been considered.

This study presents some limitations that must be taken into consideration. This is an observational single centre retrospective study, which can confer some risk of bias and limit the size of data available for analysis. The small number of patients with T2D relapse and never remission at 10 years after RYBG can overestimate the scores’ accuracy, whereby there is a need to validate our findings in different and larger patient cohorts.

In conclusion, RYGB is associated with a high rate of long-term T2D remission that can be estimated by the use of prediction scores. Among, DiaRem, Ad-DiaRem, 5y-Ad-DiaRem and DiaBetter scores, the 5y-Ad-DiaRem was the one that demonstrated to have the highest accuracy to predict long-term T2D remission after RYGB surgery. Nevertheless, the accuracy of the available scores to predict T2D remission in the long term is considerably decreased over the timespan after surgery. Thus, a better scoring system for predicting long-term T2D remission after bariatric surgery is still needed.

## Data Availability

The datasets generated during and/or analysed during the current study are available from the corresponding author on reasonable request.
